# Determination of Fracture Mechanic Parameters of Concretes Based on Cement Matrix Enhanced by Fly Ash and Nano-Silica

**DOI:** 10.3390/ma17174230

**Published:** 2024-08-27

**Authors:** Grzegorz Ludwik Golewski

**Affiliations:** Department of Structural Engineering, Faculty of Civil Engineering and Architecture, Lublin University of Technology, Nadbystrzycka 40 Str., 20-618 Lublin, Poland; g.golewski@pollub.pl; Tel.: +48-81-5384394; Fax: +48-81-5384390

**Keywords:** cementitious concrete, fly ash, nano-silica, compressive strength, splitting tensile strength, critical stress intensity factor, critical crack tip opening displacement, critical unit work of failure

## Abstract

This study presents test results and deep discussion regarding measurements of the fracture toughness of new concrete composites based on ternary blended cements (TCs). A composition of the most commonly used mineral additive (i.e., fly ash (FA)) in combination with nano-silica (NS) has been proposed as a partial replacement of the ordinary Portland cement (OPC) binder. The novelty of this article is related to the fact that ordinary concretes with FA + NS additives are most often used in construction practice, and there is a decided lack of fracture toughness test results concerning these materials. Therefore, in order to fill this gap in the literature, an extensive evaluation of the fracture mechanic parameters of TC was carried out. Four series of concretes were created, one of which was the reference concrete (REF), and the remaining three were TCs. The effect of a constant content of 5% NS and various FA contents, such as 0, 15%, and 25% wt., as a partial replacement of cement was studied. The parameters of the linear and nonlinear fracture mechanics were analyzed in this study (i.e., the critical stress intensity factor ($K_{Ic}^{S}$), critical crack tip opening displacement (*CTOD*_c_), and critical unit work of failure (*J*_Ic_)). In addition, the main mechanical parameters (i.e., the compressive strength (*f*_cm_) and splitting tensile strength *(f*_ctm_)) were evaluated. Based on the studies, it was found that the addition of 5% NS without FA increased the strength and fracture parameters of the concrete by approximately 20%. On the other hand, supplementing the composition of the binder with 5% NS in combination with the 15% FA additive caused an increase in all mechanical parameters by approximately another 20%. However, an increase in the FA content in the concrete mix of another 10% caused a smaller increase in all analyzed factors (i.e., by approximately 10%) compared with a composite with the addition of the NS modifier only. In addition, from an ecological point of view, by utilizing fine waste FA particles combined with extremely fine particles of NS to produce ordinary concretes, the demand for OPC can be reduced, thereby lowering CO_2_ emissions. Hence, the findings of this research hold practical importance for the future application of such materials in the development of green concretes.

## 1. Introduction

### 1.1. Literature Review

Concrete is one of the most commonly employed construction materials, owing to its easy workability, shapeable properties, and relatively low production cost. Unfortunatelly, the ordinary Portland cement (OPC) employed in concrete manufacture releases high quantities of carbon dioxide (CO_2_) and other greenhouse gas (GHG) emissions into the atmosphere during production. In addition, high amounts of energy, both electrical and thermal, are consumed during OPC production [1,2]. These two phenomena disrupt the balance of nature and cause climate change and global warming by increasing the temperature in the atmosphere. Due to these reasons, measures have been in place for some 30 years now to reduce and minimize the adverse effects resulting from the production of cement binder [3,4].

Therefore, in order to reduce OPC production, researchers have turned to studies on materials which can replace non-ecological OPC in the composition of a concrete mix [5]. Thus, it should be noted that at present, the substitutes for cementitious binder are mostly pozzolanically or hydraulically active mineral materials. These include but are not limited to materials such as siliceous and calcareous fly ashes (FAs), silica fume (SF), ground granulated blast furnace slag (GGBFS), and other useful mineral additives [6].

These alternative binder materials have two important advantages. First of all, they are able to catalyze the chemical processes involved in the formation of the cement’s matrix structure [7,8]. In addition, concretes made with their participation are environmentally sustainable [9].

In general, materials which can replace OPCs in a concrete mix are referred to as supplementary cementitious materials (SCMs) [10,11]. On the other hand, their participation forms concrete composites with the advantages of being ecological, economical, useful, and valuable [12].

For many years, the most valuable and most widely used SCM in the cement and concrete industry has been FA [13,14]. This is due to the fact that concrete made with the addition of this industrial waste has the four beneficial properties mentioned above. In addition, the fine and pozzolanically active FA grains are able to strengthen and densify the structure of a concrete composite, which results in improvement in many of its characteristics and parameters [15,16].

For instance, in mature concretes containing up to 20% coal FA, improvements include their following features: the physical and strength parameters, fracture toughness [17], resistance to dynamic and impact loads [18], homogenization of the material structure, and reduction in the size of interfacial microcracks [19]. In addition, in earlier studies of the microstructure of concrete containing a 20% FA additive, it was found that this material had a compact structure and extremely small crack widths (of about 700 nm) in the interfacial transition zone (ITZ) between the coarse aggregate grains and the paste. This composite also contained mainly fibrous C-S-H phases of type (I), C-S-H phase type (II) in the form of a honeycomb structure, and C-S-H phase type (IV) in the form of spherical agglomerates, but with a much lower intensity than that in the phase of type (I).

Unfortunately, in addition to the numerous advantages which the substitution of OPC with FA in the composition of a concrete mix brings, composites made in this way contain one significant and extremely important disadvantage from the utilitarian point of view. The problem under discussion concerns the slowing down of curing processes in the cement matrix containing an FA additive [20]. According to [21], FA delays the early hydration of cement by introducing aluminum and organic ions derived from FA dissolution into the liquid phase of the paste. The magnitude of the delay increases in proportion to the share of FA in the binder composition [22].

In order to prevent these negative effects on the use of FA in a concrete structure, to date, a hybrid combination of two or three pozzolanic additives has most often been used. Silica fume was used most often for this purpose. Such measures made it possible to reduce the negative effects associated with the reduction in the values of the mechanical parameters of fly ash concretes at an early age. Based on previous studies, it was observed that the use of SF in FA concretes contributed to a substantial increase in the fracture toughness of these materials at an early age.

However, currently, concrete technology is increasingly using materials which exploit the advantages of nanotechnology to improve the properties of immature concretes incorporating FA additives. The advantages associated with these activities were described in [23], whereas the most frequently used nanomaterials applied to strengthen the structures of concrete composites containing FA are collected in Table 1.

Previously, the combined effect of nanomaterials in combination with different SCMs was also investigated [32,33]. During these studies, the properties of concretes manufactured with a binder containing an FA additive, in conjunction with different types of nanomaterials such as nano-SiO_2_, nano-Al_2_O_3_, nano-TiO_2_, and nano-CaCO_3_, were studied [34,35,36,37,38,39,40,41,42,43,44,45,46]. Based on previously conducted research, it was established that in most cases, the improvement in the parameters of concrete composites is positively influenced by the total addition of FA up to 30%, in combination with a nanoadditive dose up to the amount of 5% [47,48]. This combination of both materials also significantly improves the properties of the cementitious composites at an early age, as well as after long periods of curing [49,50,51].

Of the wide group of nanomaterials listed above, the oxide most often used to actively modify the structure of cementitious composites is nano-SiO_2_, or nanosilica (NS). It is the most widely produced nanomaterial in the world, and it is significant that NS production has increased from about 100,000 tons in 2010 to about 400,000 tons in 2020. Due to its favorable properties, this material is being used in both industrial applications and scientific investigations [52].

Nanosilica is also widely used in the cement and concrete industry [53]. The multiplicative nature of the interactions of active and ultrafine NS particles causes them to be applied alone as a nanoadditive [54] or in combination with other active SCMs [55].

Such significant use of this useful material is due to the fact that composites with NS added [56,57,58,59,60,61,62,63,64] achieve high values for their mechanical and fracture toughness parameters, and these concretes are characterized by increased durability. In addition, such materials have increased resistance to static, cyclic, fatigue, and dynamic loads as well as to the diffusion of harmful substances deep into the structure of the composite. In terms of the scientific topic undertaken, it should also be stated that NS is able to reduce, to some extent, the delay in the processes associated with the formation of the early composite structures in concretes with fly ash [61,62].

**Figure 1:**
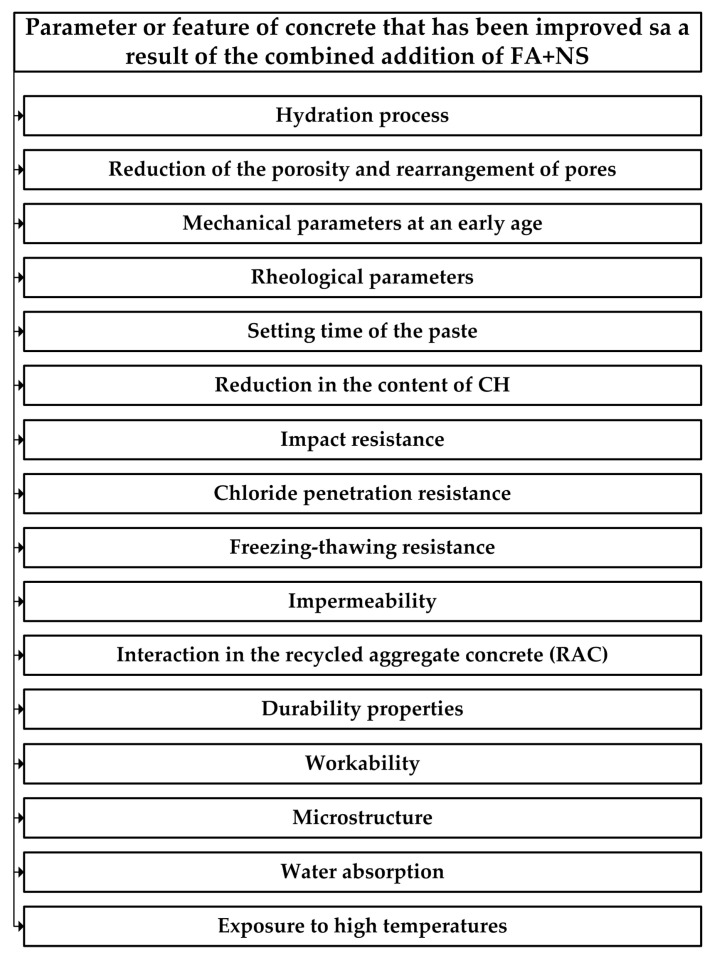
Beneficial properties of concretes with the addition of FA and NS [63,64,65,66,67,68,69,70,71,72,73].

For these reasons, numerous experiments have been carried out to date to evaluate the properties of cementitious composites based on the binder in the OPC + FA + NS system. Based on the results of previous studies, Figure 1 summarizes the mechanical, physical, and rheological parameters of cementitious composites as well as their structural characteristics, which were improved by the application of addition a combination of FA and NS.

Unfortunately, although most of the important properties and features of cementitious composites based on ternary binders containing FA and NS have been analyzed to date, the range of experiments to date does not fill all the gaps in this topic. In fact, in the world’s literature, one can find only a few works presenting the results of studies on the fracture toughness of ordinary concretes containing FA + NS. However, this topic is quite important due to the close relation between the susceptibility to cracking of concretes, their durability, and consequently the time range after which the concrete structure will require repair.

Aside from this, experiments on the fracture toughness of concretes with FA or FA in combination with other pozzolanic materials (mainly SF) have already been the subject of previous research. Thanks to the results of these studies, it has been established, for instance, that the optimal amount of FA in the context of improving fracture toughness (i.e., critical stress intensity factor ($K_{Ic}^{S}$) of the fly ash concrete), the influence of the w/c ratio, and its influence regarding SF content on the fracture mechanic parameters of $K_{Ic}^{S}$ and the critical crack tip opening displacement (*CTOD*_c_) of gravel concrete containing SF, as well as the positive influence of both pozzolanic active mineral additives (i.e., FA + SF) on delays in the fracture processes in ordinary immature and mature concrete [74].

Unfortunately, with regard to composites based on ternary binders involving FA and NS, studies on fracture toughness are severely limited. In addition, they have thus far been carried out only on high-strength concretes or concretes with a high volume of FA [50]. These experiments have focused on, for example, finding relations between the water-to-binder ratio (w/b) and the fracture toughness of such composites, as well as the determination of the percentage of NS which is beneficial from the point of view of improving the fracture mechanic parameters of such composites [75,76]. Based on the results obtained from these experiments, it was established that improvement of the fracture toughness of these types of concrete is influenced by reducing the w/b ratio to 0.35 and the addition of NS at levels of up to 5% of the cement mass [75,76,77,78]. However, given that ordinary concretes with FA + NS additives are most often used in construction practice, and there is a decided lack of fracture toughness test results concerning these materials, it was decided to fill this gap in the literature. For this purpose, a wide range of laboratory tests were carried out. Over the course of them, the basic parameters of the fracture mechanics were studied for the test elements loaded at a mode I fracture. The fracture toughness was estimated on the basis of the fracture mechanic parameters characterizing the fracture processes of the material in both linear and nonlinear terms. In addition, the effect of the used combinations of the two SCMs on the basic strength parameters of the concretes was also analyzed.

After conducting the experimental tests, an in-depth statistical analysis of the obtained results was carried out according to the assumptions described in Section 3.1. For this purpose, the values of significant statistical factors such as the standard deviations (δ) and coefficients of variation (ν) were studied. The distribution of the extreme values of each tested mechanical parameter was also analyzed. With these detailed analyses of the obtained test results, it was possible to obtain insight into the optimal combination of FA in conjunction with NS which, with respect to ordinary cementitious concretes, causes a delay in the destructive processes in concrete elements and a significant increase in the fracture toughness of the material.

In addition, after determining the values of the analyzed parameters of the fracture mechanics, an attempt was also made to relate the results obtained to conclusions drawn from earlier microstructural studies on the same composites. This is because on the basis of these studies, it was established that OPC substitution with a combination of active pozzolan SCMs in the form of FA + NS also brings clear benefits in improvement to the cement matrix morphology of modified composites. In previous studies, it was found that the combined usage of NS and FA has synergistic and positive effects on improving the microstructures in such concretes. For instance, it was observed that due to the activation of FA grains by extremely fine and highly active NS particles, voids, pores, and initial microcracks in the structure of a cement matrix are filled. Hence, homogenization of the cement matrix effectively improves the microstructure arrangement of composites incorporating FA and NS [79]. Therefore, it seems that such beneficial effects of strengthening the material structure should also have a measurable effect on improving their fracture toughness. We attempted to demonstrate this in our own undertaken research.

### 1.2. Theoretical Background

To evaluate the strength of concrete elements containing defects, knowledge of the material characteristics which take into account the stress distribution in the area of the initial crack tip is required. The basic fracture criterion for concrete composites is the local force fracture criterion of the linear-elastic fracture mechanics. It uses the concept of the so-called stress intensity coefficient. This parameter takes into account the description of stress fields around the crack tip, and its value depends on the loads acting on the element, crack dimensions, and specimen size. The critical value of the stress intensity factor describes the state beyond which the process of its destruction in the material can begin. In this case, the initial crack will begin to develop in an uncontrolled manner. When the material is dominated by tensile stresses (i.e., there is a so-called mode I fracture), the critical stress intensity factor is defined as *K*_Ic_ [80,81].

However, the stress intensity factor refers to the description of the stress state at the tip of the initial crack in completely brittle materials, where there are no zones of plasticity in the crack tip area. In contrast, the failure of some types of concrete composites often bears the hallmarks of so-called quasi-plastic fractures [82,83].

Such a phenomenon is associated with the appearance of micro-cracks in the area of the initial crack and the rotation and separation of coarse aggregate grains from the cement matrix. The signs of quasi-plastic behavior in the fracture process are also observed in concretes containing some SCMs or additives of other types. Such a phenomenon is seen, for example, in concretes containing FA or composites with rubber crumbs [84].

The formation of some small plastic deformations before crack propagation is allowed when applying elasto-plastic theory (i.e., nonlinear fracture mechanics). The fracture mechanic parameters which take into account the elasto-plastic model of the material were proposed by J. R. Rice to be the critical unit work of failure *J*_Ic_ [85] and critical crack tip opening displacement *CTOD*_c_ [86].

Therefore, in order to accurately characterize the fracture toughness of a new type of concrete composite reinforced by both FA and NS, this paper provides test results and scientific discussion in terms of the implemented substitution of OPC with FA and NS as well as analyses of the effect of this solution on the fracture mechanic parameters of concretes in both linear and nonlinear terms (i.e., *K*_Ic_, *J*_Ic_, and *CTOD*_c_). It should be noted that additional detailed explanations of the characteristics and the significance of all fracture mechanics parameter considered in the analyses are given in the source monographs and publications on the topic (e.g., [87]).

## 2. Experimental Investigation

### 2.1. Framework Research Program

The essential task of the proposed examinations was to assess in ordinary concretes, based on a cement matrix enhanced by FA and NS, the fracture mechanic parameters (i.e., the critical stress intensity factor $K_{Ic}^{S}$, critical crack tip opening displacement *CTOD*_c_, and critical unit work of failure *J*_Ic_).

In addition, the effect of the used combinations of the two SCMs on the basic strength parameters of the concretes was also analyzed (i.e., the compressive strength *f*_cm_ and splitting tensile strength *f*_ctm_).

The plan for the experiment included assessing the impact of modifying the cementitious binder with a combination of NS and FA on the parameters of cementitious ternary concretes (TCs). All composites were formed according to the same procedures described in the following subsections. The basic binder used to make the samples was OPC, while the constant parameter was the water-to-binder ratio (w/b = 0.4). The variable parameters adopted in the experiment were the used amounts of the binder modifiers. It should be noted that in each series of concrete, OPC was substituted with additives by weight. However, the tested concrete variables assessed during the experiments were the strength parameters and the basic parameter of the fracture mechanics. The detailed plan of all planned experiments is summarized in Table 2. 

All mechanical parameters summarized in Table 2 were assessed after 28 days of specimen curing. However, it should be mentioned here that the presented research is only the first stage in assessing the combined impact of FA and NS on the parameters of the fracture mechanics of cementitious concrete. Due to the fact that the addition of FA slows down the processes related to the formation of the concrete’s composite structure to some extent, which may result in a reduction in the analyzed mechanical parameters, such tests should be conducted while taking into account influence of the material’s curing time. Therefore, in the future, we plan to carry out further series of experiments assessing the fracture toughness of the subject composites. Their schedule would include assessment of the fracture mechanic parameters of concretes reinforced with FA and NS in two different time intervals, namely at an early age (i.e., up to the 28th day of specimen curing) and in the period over the mature age (i.e., from the 28th to 365th day since the specimens were prepared).

### 2.2. Materials

The OPC CEM I 32.5 R was used for all mixtures [88]. The main binder used in the study was manufactured by the Chełm cement plant in Poland. Additionally, the binder composition modifiers in the concrete mixtures were siliceous fly ash (FA) and nano-silica (NS). The FA used was type F, according to ASTM C618-03 [89]. This SCM was manufactured by the Puławy thermal-electric power station in Poland. However, as a nanoaddition, white powdered synthetic NS Konasil K-200 fabricated by the OCI Company Ltd. in the Seoul, Republic of Korea was used.

**Figure 2:**
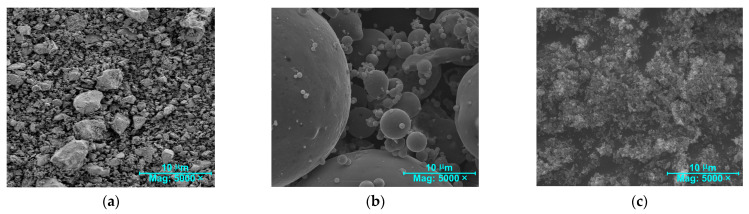
SEM micrographs of cementitious materials used in the studies: (**a**) OPC, (**b**) FA, and (**c**) NS.

The physical properties and chemical composition of the OPC, FA, and NS are illustrated in Table 3, and Figure 2 presents the SEM images of all binders used. In order to compare the particle sizes of the cementitious materials, the same scales and magnifications were used (Figure 2). Moreover, it should be noted that the chemical compositions of all binders were determined using XRF method.

On the basis of microscopic images of the mineral binders used in the research, presented in Figure 2, and the data contained in Table 1, it can be clearly stated that NS was the smallest of all the cementitious materials used. Due to the use of this nanoadditive, it was possible to effectively seal the composite structure and thus reduce its porosity. This then allowed improving the mechanical and physical parameters of the composite and increasing its tightness (i.e., increased composition and reduced porosity). This in turn implies an improvement in the durability of the material. The research in question will assess how the applied material modification improves the fracture toughness of concrete made from binary and ternary binders.

However, the FA grains were larger than the NS grains, with almost perfectly spherical shapes (Figure 2b). The cement grains, on the other hand, were the largest and had irregular shapes (Figure 2a).

In addition to cementitious materials, pit sand passing beyond 2.0 mm and with a specific gravity of 2.60 g/cm^3^ as fine aggregate and natural gravel ranging from 2.0 to 8.0 mm and with a specific gravity of 2.65 g/cm^3^ as coarse aggregate were used. The two types of aggregates used originated as crumbs from different rocks, such as quartz, limestone, granite, and gneiss. The particle size distributions of both above aggregates are presented in Table 4.

All mixtures were prepared using laboratory pipeline water free from contamination. Since NS significantly reduces the workability of concrete mixtures, as shown in [90], the chemical admixture superplasticizer (SP) STACHEMENT 2750, based on polycarboxylates, was used in this study. This helped partially minimize these negative effects and significantly improved the workability of the concrete mixtures with the addition of NS.

### 2.3. Mix Proportioning

In order to carry out the strength and fracture toughness tests, three concrete mixes containing a modified binder including OPC, FA, and NS and a reference mix containing 100% cementitious binder were manufactured. Cement paste was prepared with a water-to-binder ratio of 0.4. Six specimens were prepared for each group.

Details on the specimen preparation for each test are provided in Section 2.4. The adopted mix designs per cubic meter are detailed in Table 5. It should be noted that the contents of FA and NS were selected based on the author’s previous work [79].

### 2.4. Specimen Fabrication

The concrete mixture was prepared in a DZB-300 counter-rotating mixer with a capacity of 150 L and power of 1.1 kW. The mixing process of all mix components was a multi-stage process. At the beginning, the procedure of calculating and weighing the amount of each material needed for specimen preparations was carried out. After this preliminary step, preparation of the concrete mixture proceeded.

The preparation of the specimens for cementitious concretes involving FA and NS was performed as described below. It included four characteristic steps. As a result of these activities, the mixture necessary to fabricate concrete specimens was prepared. The procedure for concrete mixture preparation was as follows.

First, coarse aggregate and fine aggregate were dry-mixed for 60 s. Then, the mixer was soaked with water. After the mixer was wetted, the coarse and fine aggregates were added and stirred for the next 60 s, followed by stirring with OPC and FA for 180 s. In the meantime, SP and NS particles were added to half of the water content in order to pre-mix them for 30 s. Next, this mixture was added to the mixer container and mixed for 90 s. In the next step, the remaining amount of water was added. At the end, final mixing of all of the used concrete mix components was performed for a period of 120 s in order to obtain a homogeneous mass of the mixture.

After preparation of the concrete mixture, formation of the specimens and the subsequent processes which enabled the specimens to be used in the experiments proceeded. For this purpose, plastic and wooden molds for casting of the specimens were prepared. Then, the process of molding the specimens was carried out, and they were cured wet after fabrication.

All specimens were demolded after 48 h and cured under a water curing tank for the first 14 days (t = 20 ± 2 °C, RH = 95–100%). For the next 2 weeks, the specimens were cured in laboratory conditions (t = 20 ± 2 °C, RH = 40%) and then examined 28 days after their preparation.

### 2.5. Equipment Settings and Test Procedures

#### 2.5.1. Mechanical Parameter Testing

The testing of the main strength characteristics of the analyzed composites (i.e., *f*_cm_ and *f*_ctm_) was carried out on a Walter + Bai ag type NS19/PA1 testing machine with a maximum capacity of 3000 kN and with the application of cubic specimens (150 mm).

The experiments were carried out under static loading. The following loading rates for the specimens were adopted:

- From 0.5 to 0.8 MPa/s during the compressive strength test;
- From 0.06 to 0.04 MPa/s during the splitting tensile strength test.

Six cubic specimens with sides of 150 mm were prepared for each mixture ratio and for both mechanical tests. 

The following standards were applied to conduct the strength tests:

- EN 12390-3: 2011+AC: 2012 [91] for the compressive strength test;
- EN 12390-6: 2009 [92] for the splitting tensile strength test.

#### 2.5.2. Fracture Toughness Evaluation

**Figure 3:**
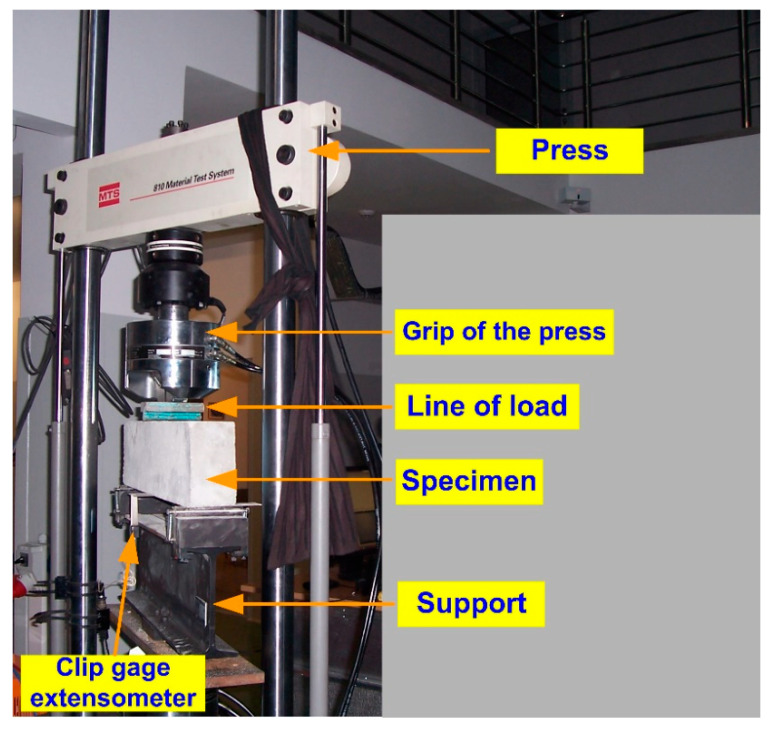
A view of the test stand with full instrumentation.

The study of the parameters of the fracture mechanics of concretes in the first model of cracking was carried out on an MTS 810 testing press. The loads in the specimens were applied in an incremental manner. The process of development of initial cracks was also evaluated during the study. For this purpose, a special clip gauge extensometer (632.03 F-3 type) was used. During the experiments, the work of the sensor was correlated with the values of changing loads applied to the MTS 810 press. The whole experimental stand with full instrumentation is shown in Figure 3.

The results were obtained from the records of the MTS 810 testing press, whereas during the experiments, the following relationships were recorded for each sample:

- Load (*F*) crack mouth opening displacement (*CMOD*) *F–CMOD*;
- Load (*F*) deflection of the specimen (*D*) *F–D*.

Based on the knowledge of the *F–CMOD* relationships, it was possible to calculate the fracture mechanics parameters $K_{Ic}^{S}$ and *CTOD*_c_ later [93,94]. However, based on the *F–D* curves, it was possible to determine the values of *J*_Ic_ [92].

All specimens were successively loaded and unloaded from a level of 0 kN to the values of the forces, causing their destruction. Thus, it was possible to estimate the effect of incremental loads on their failure process [93,94].

**Figure 4:**
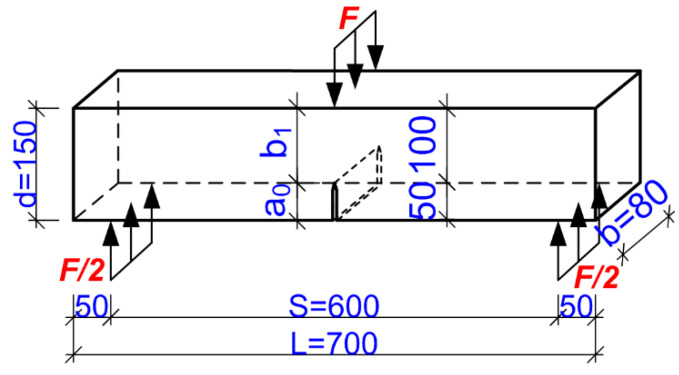
A schematic of the specimen used in the studies, with dimensions in millimeters.

A fracture toughness test was carried out in accordance with the RILEM draft recommendations TC-89 FMT [95]. Six 80 mm × 150 mm × 700 mm beam specimens with one initial central crack of 5 mm × 80 mm × 50 mm were prepared for each mixture ratio. A schematic of the specimen, along with its dimensions and the relevant data needed to determine the fracture mechanic parameters, is shown in Figure 4.

On the basis of the results obtained from experimental tests and the data taken from the *F–D* and *F–CMOD* diagrams, the basic parameters of the fracture mechanics of the analyzed composites in terms of both their linear and nonlinear fracture mechanics were determined.

The fracture mechanic parameters $K_{Ic}^{S}$ and *CTOD*_c_ were determined from the relationships given in [95]. The necessary equations for determining these parameters are summarized below:

(1) $$K_{Ic}^{S}=3\left(F_{max}+0.5W\right)\frac{S{\left(\pi a_{c}\right)}^{1/2}F\left(\alpha \right)}{2W^{2}b},$$

in which

$$F\left(\alpha \right)=\frac{1.99-\alpha \left(1-\alpha \right)\left(2.15-3.93\alpha +2.7\alpha^{2}\right)}{\sqrt{\pi^{1/2}\left(1+2\alpha \right){\left(1-\alpha \right)}^{3/2}}},$$

where $\alpha =\frac{a_{c}}{d};\;F_{max}$ is the measured maximum load; *W*$=\frac{W_{0}S}{L};$ and $W_{0}$ is the self-weight of the beams *d*, *b*, *S*, and *L* according to Figure 3:

(2) $${CTOD}_{c}=\frac{6F_{max}Sa_{c}V_{1}\left(\alpha \right)}{Ed^{2}b}{\left[\left(1-\beta^{2}\right)+\left(1.081-1.149\alpha \right)\left(\beta -\beta^{2}\right)\right]}^{1/2},$$

Here, $\alpha =\frac{a_{c\;}}{d},\;\beta =\frac{a_{0\;}}{a_{c}},and\;a_{0}$ is the initial notch depth according to Figure 4. On the other hand, the critical values of *J*_Ic_ were estimated according to the ASTM E 1820-01 standard [96], based on Equation (3):

(3) $$J_{Ic}=\frac{A}{2bb_{1}}$$

where *A* is the energy absorbed in the specimen up to the moment of the initial crack growth. This energy can be calculated by finding the area (i.e., by taking the integral) underneath the *F–D* curve to the force *F*_max_ for *b* and *b*_1_ according to Figure 4.

## 3. Results and Analysis

### 3.1. The Range of Obtained Test Results and Their Statistical Parameters Compiled for Further Analysis

During the development of experimental results, for both the strength and fracture mechanic parameters, the average values of six specimens for each test were considered. Then, an in-depth statistical analysis of the obtained test results was carried out. Through its course, significant statistical parameters were determined for each type of experiment and each type of composite. In addition, the summaries of the results also include the obtained extreme values from the conducted tests. For better clarity, all of the above data for each parameter analyzed are summarized in separate tables. Each table contains the following detailed specifications for each type of experiment:

- The average values of the analyzed mechanical parameters: $\bar{f_{cm}}$, $\bar{f_{ctm}}$, $\bar{K_{Ic}^{S}}$, $\bar{{CTOD}_{c}}$, and $\bar{J_{Ic}}$;
- The percentage of relative changes of a given parameter for the series of concrete modified by FA and NS with regard to the results obtained for the reference concrete;
- Statistical parameters, including the standard deviation δ and coefficient of variation ν;
- The maximum (max.) and minimum (min.) values of each parameter obtained during the studies.

### 3.2. Strength Parameters

The evaluated mechanical parameters of the analyzed concretes (i.e., *f*_cm_ and *f*_ctm_) are shown with their statistical factors in Table 6 and Table 7, respectively.

When evaluating the strength parameter values of concretes based on binders containing FA and NS, it is clear that the proposed solution had a significantly positive impact on the obtained test results. However, the values of *f*_cm_ and *f*_ctm_ varied depending on the percentage of FA and NS, which replaced OPC in the concrete mix composition.

Based on the results presented in both tables, it is clearly visible that the most favorable modification effect was achieved in the case of the composite based on a ternary binder in which OPC was replaced with 5% NS additive and 15% FA additive. Increasing the amount of FA in the concrete mix from 15 to 25% resulted in a slight decrease in the analyzed strength parameters. The least visible improvement effect for both *f*_cm_ and *f*_ctm_ was observed in the case of concrete containing only active NS particles. Nevertheless, in the case of the TC-1 series concrete, the values of the strength parameters were also clearly higher compared with the results obtained for the reference concrete (Table 6 and Table 7). 

Thorough analysis of the percentage changes in *f*_cm_ and *f*_ctm_, showed the following in the cases of the TC-1, TC-2, and TC-3 concretes:

- The *f*_cm_ increased by 18%, 38%, and 29% compared with the results obtained for the unmodified concrete, respectively;
- The *f*_ctm_ increased by 17%, 36%, and 28% compared with the results obtained for the reference concrete, respectively.

### 3.3. Fracture Toughness

**Figure 5:**
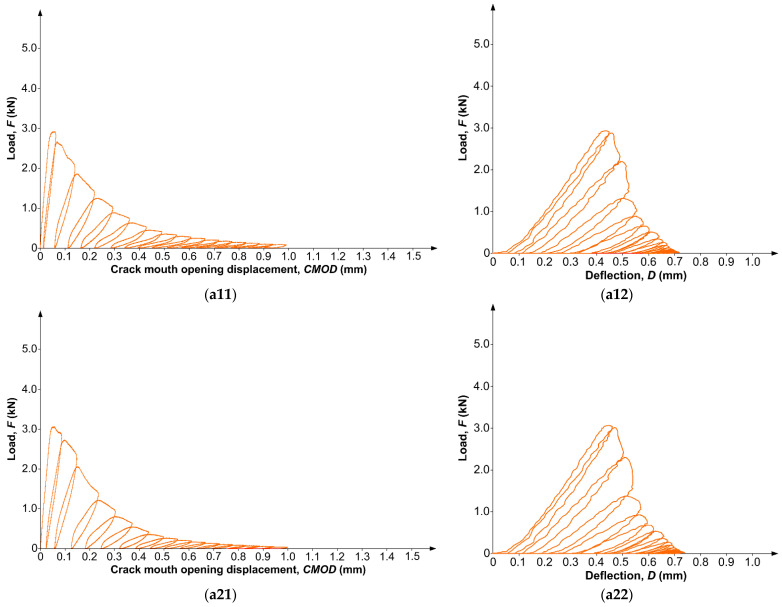

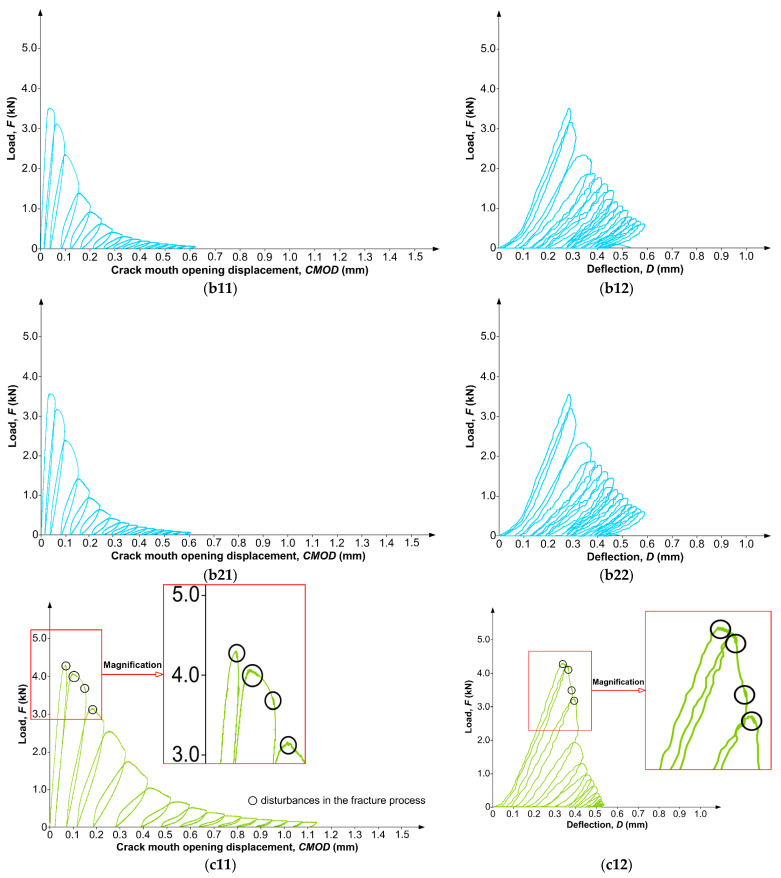

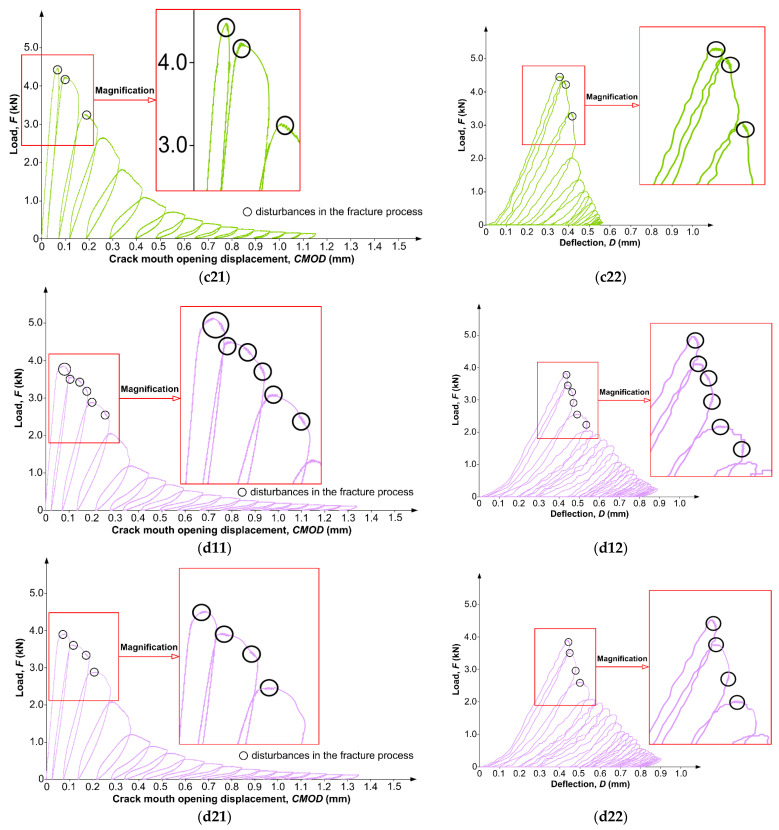
Examples of *F*–*CMOD* (1) and *F*–*D* (2) relationships of analyzed composites: (**a**) REF, (**b**) TC-1, (**c**) TC-2, and (**d**) TC-3; description in the text.

Figure 5 shows examples of the *F–CMOD* and *F–D* relationships for all series of analyzed composites. For each batch of concrete, two exemplary representative graphs of both analyzed relationships were complied. The charts of both relationships for each material are summarized in pairs. In addition, the charts of concretes containing the simultaneous addition of FA and NS (i.e., the TC-2 and TC-3 series) also mark the unusual disturbances which occurred during the progressive fracture process of the specimens of these concretes. In order to better visualize the observed effect, Figure 5 shows the exact magnifications of this phenomenon. 

All the charts in Figure 5 are marked with two digits. The first digit indicates the number of the graph in a given series of concrete, while the second digit indicates the type of graph. The individual series of concrete are marked as follows: (a) REF, (b) TC-1, (c) TC-2, (d) TC-3. On the other hand, *F–CMOD* charts are marked with a (1), whereas *F–D* charts are marked with a (2). For instance, the designation of chart (a21) indicates that this is a second example of the *F–CMOD* chart for REF series of concrete.

A thorough evaluation of the graphs of the two analyzed relationships (i.e., *F*–*CMOD* and *F*–*D*) was aimed at showing the differences which occurred in the process of fracture and failure of beams made of four different materials. When comparing the graphs, the following features were mainly taken into account: the values of the *F*_max_ forces causing the development of the initial crack, the characteristics of the *F*–*CMOD* and *F*–*D* graphs in terms of their shape, inclination, and the number of fatigue cycles leading to the destruction of specimens, the values of *CMOD* at the moment of failure of the beams, and the length of the fracture process of the beams (i.e., from initiation of the initial crack until the phase of complete failure of the specimens).

Based on the conducted observations, the following characteristic features of the fracture behavior of specimens made of different materials were established, summarized in Table 8.

In addition, by carefully analyzing the *F*–*CMOD* and *F–D* curves for all types of concrete, it was found that reinforcement of the cement matrix with FA particles in combination with NS nanoparticles caused additional subtle differences in the fracture processes of such materials. This may be related to earlier observations of the fracture processes of fly ash concretes. This is because in previous studies, it was observed that the addition of FA to concrete composite had a clear effect on changes in the width, shape, and trajectory of the observed cracks.

The most significant features observed in the behavior of individual concretes during their progressive deterioration process are characterized below:

- For the REF concrete, the lowest average values of the *F*_max_ forces and the lowest *CMOD* at a beam’s failure were recorded. The *F*–*CMOD* and *F–D* graphs were slightly inclined, whereas the behavior of the concrete samples in the fracture process was similar to that of quasi-plastic destruction. The average number of fatigue cycles for this material ranged from 13 to 15. It was observed that the entire fracture process of the specimens was quite short compared with the behavior of the beams made of other concretes, mainly in relation to the TC-2 and TC-3 series of concretes (Figure 5a, Table 8).
- For the TC-1 series concrete, the fracture process of the specimens was quite short (the number of loading cycles for this material ranged from 12 to 14), while the *F*_max_ forces were about 20% higher than those in the results obtained for the reference concrete. On the other hand, the specimens of this series of concrete were clearly brittle and damaged at much lower *CMOD* values than in the case of the REF concrete. Both failure curves of the specimens, presented in Figure 5b, were slender in the first phases of their loading. Only in the case of this material were no yielding effects observed in the fracture process of the beams (Figure 5b, Table 8).

It should be noted that a similar behavior to that of the samples during the fracture toughness tests under mode I fracturing was observed in the case of concretes based on a ternary binder modified with NS in conjunction with SF [97].

- For the TC-2 series concrete, the highest values of the maximum forces transmitted by the samples among the results obtained for all the tested concretes were recorded. In addition, it should be noted that these forces reached values higher than the results observed for the reference concrete by 40%. The fracture process of the specimens made of this concrete was slightly elongated, and the number of loading cycles was from 14 to 16. Slight signs of quasi-plasticity were observed in the fracture process of the specimens. However, the destruction of the beams occurred at much higher *CMOD* values than in the case of the TC-1 series concrete. In addition, in the *F–CMOD* as well as *F–D* charts, signs of heterogeneity of the material were visible in the behavior of the samples during cracking. Moreover, both curves showed additional minor micro-damage in the area of the propagating crack. In the load-unload graphs, this phenomenon appeared with the occurrence of small additional disturbances. These effects, which are marked on both curves in black circles, were more visible in the first fatigue loops and were emphasized in the cases of both the TC-2 series composite and concrete with a higher FA content (i.e., the TC-3 series). The observed phenomenon was probably the result of the presence of FA grains which had not completely reacted or only partially reacted in the structures of concretes with ternary binders. This caused the damage development processes in this part of the composite to disperse beyond the main fracture zone. The external energy transferred from the press in the form of an external load was concentrated in these samples both on their weakest areas (i.e., an artificially modeled crack) and on places with heterogeneous structures. This in turn resulted in the appearance of additional microcracks and, consequently, the occurrence of delicate micro-peaks on the *F*–*CMOD* and *F*–*D* graphs (Figure 5c, Table 8). It should be noted that the phenomenon of step nonlinearity visible on the destruction curves was not observed in the tests of the other two composites (i.e., the REF and TC-1 series).

It should be noted, however, that similar behavior to the samples in the process of cracking was observed, among others, in testing concrete based on quaternary binders. On the other hand, other details of such behavior by composites with matrices of an OPC + FA + NS arrangement are described in [97].

- For the T3 series concrete, the recorded *F*_max_ forces were only 30% higher compared with the values obtained for the reference concrete. Thus, it can be concluded that increasing the FA content of the concrete mix resulted in a decrease in the composite’s ability to minimize its susceptibility to cracking during the progressive loading process. In addition, the fracture process of this material was clearly elongated, with an average number of fatigue cycles in the range of 15–18. The *F*–*CMOD* and *F–D* diagrams for the specimens made of this concrete are clearly sloped. In addition, both graphs show a clear effect of the flow of the material, which indicated a clearly quasi-plastic method for its destruction (Figure 5d, Table 8).

However, the readings of the *CMOD* parameter at the final stage of loading the specimens were the highest among the values obtained for all tested composites. Moreover, as in the case of the TC-2 series concrete, additional unusual micro-disturbances were also visible in both graphs assessing the process of development of the initial crack. They indicated the development of fracture processes in this material in a non-standard way and the occurrence of probable, clear heterogeneity [98].

In addition, by carefully analyzing for all concretes their features, such as the shapes of the *F*–*CMOD* and *F–D* diagrams, the duration of the fracture processes in each material, and the obtained average values of the *F*_max_ and *CMOD* parameters, one can clearly see the effect of the material modification applied on the level of their brittleness. The most brittle one turned out to be the concrete of the TC-1 series. On the other hand, the addition of FA caused the occurrence of quasi-plastic phenomena in the fracture process of concretes of the TC-2 and TC-3 series. This was more clearly noticeable in the case of concrete with a higher content of FA (Figure 5). Such phenomenon occurring in concretes with added FA was also confirmed by the results of other studies [56].

At this point, it should also be added that a detailed analysis of the fracture processes of concretes reinforced by FA and NS was presented in [56]. In this work, the behavior of each composite in the process of its deterioration and destruction was studied in detail. A precise digital image correlation technique was used for this purpose.

In addition to evaluating the fracture behavior of individual composites reinforced by FA and NS, *F–CMOD* and *F–D* plots also allowed determination of the fracture mechanic parameters in linear and nonlinear terms. The results obtained from these calculations are summarized in Table 9, Table 10 and Table 11.

Based on the data presented in the above tables, it can be strongly concluded that, as in the case of the strength parameters, with regard to the fracture toughness results, the effect of the applied modification was evident. All of the three analyzed fracture mechanic parameters (i.e., ${\;K}_{Ic}^{S}$, *CTOD*_c_, and *J*_Ic_) increased significantly. They amounted, in comparison with the reference concrete, to levels ranging from almost 20% for the concrete of the TC-1 series (i.e., with only 5% NS) to almost 40% for the concrete with a combined addition of 5% NS and 15% FA.

Thorough analysis of the percentage changes in $K_{Ic}^{S}$, *CTOD*_c_, and *J*_Ic_, presented in Table 9, Table 10 and Table 11, showed the following in the cases of the TC-1, TC-2, and TC-3 concretes:

- $K_{Ic}^{S}$ increased by 19%, 38%, and 29% compared with the result obtained for the REF concrete, respectively;
- *CTOD*_c_ increased by 19%, 38%, and 30% compared with the result obtained for the concrete without additives, respectively;
- *J*_Ic_ increased by 18%, 36%, and 28% compared with the result obtained for the concrete without additives, respectively.

When looking at the clear percentage differences in the values of the fracture mechanic parameters of the concretes reinforced with two active SCMs (i.e., FA and NS), it can be concluded that they were the result of the synergistic interaction of these two materials in the structure of the cement matrix. The pozzolanically active FA grains activated with ultrafine NS particles caused additional homogenization and strengthening of the cement matrix [98]. As a result, the fracture mechanic parameters, both in linear and non-linear terms, reached significantly higher values in these concretes.

Similar benefits from using a combination of FA and NS in terms of improving the fracture toughness of cementitious composites were also observed for fiber-reinforced high-volume fly ash cement mortar [56]. The reasons for such beneficial effects from the additives used will be discussed in Section 4. 

## 4. Discussion

Both the analysis of the results evaluating the strength parameters of the concretes and the values of the fracture mechanic parameters, in linear and non-linear terms, clearly indicate the benefits of using a combination of FA and NS when modifying the composition of a cementitious binder. This was evidenced by the fact that the most favorable effect of strengthening the concrete structure associated with a marked improvement in all analyzed mechanical parameters of the composites was observed in the concretes of the TC-2 and TC-3 series. This trend was also confirmed by the results of other previous studies [99]. They showed that FA grains to the amount of up to 20%, combined with NS particles to the amount of up to 5%, caused the formation of additional C-S-H phase products in the structure of a cement matrix. In turn, the matrix of such a composite becomes more compact and less porous. Thus, the mechanical parameters of such concretes also increase. This is confirmed by the results in Table 6, Table 7, Table 9, Table 10 and Table 11.

The above-mentioned effect of the marked improvement in the properties of concretes of this type was mainly due to the mutual interaction of fine and active FA and NS particles. At the microstructural level, the synergy between the two materials is a result of the fact that the highly pozzolanically active NS particles strongly activate the formation of additional C-S-H phases on the slightly less pozzolanically active FA grains. As a result of these interactions, the final matrix structure in concretes based on ternary binders is made up of as many as three components. The individual matrix segments, on the other hand, are the result of the following subtle chemical reactions: hydration and hydrolysis reactions of the basic binder (i.e., OPC), the typical reaction of FA grains in the presence of OPC, resulting in an increase in the C-S-H phase due to reduction in the CH phase, and the occurrence of excess phases, mainly C-S-H, resulting from stimulation of less reactive FA grains by highly reactive NS nanoparticles.

However, the higher content of FA grains in the composition of the binder, which are partially unreacted, causes a slight reduction in the final strengthening effect of the cement matrix structure [67,68,69,90,100,101]. Therefore, in the case of concrete of the TC-2 series, the increase in all analyzed mechanical parameters was at a slightly lower level (Table 6, Table 7, Table 9, Table 10 and Table 11).

On the other hand, the lowest improvement in the values of the analyzed strength and fracture mechanic parameters was obtained for the composite reinforced only by NS. As previous microscopic studies have shown, the matrix in concretes of this type is less compact and has a higher number of structural microcracks than in concretes based on OPC + FA + NS binder compositions. This causes the internal structure of such composites to be less homogenized [85]. Consequently, this resulted in a slightly smaller strengthening effect of the concrete of the TC-1 series and a limited increase in the analyzed mechanical parameters of this material (Table 6, Table 7, Table 9, Table 10 and Table 11). On the basis on the results of comprehensive tests assessing both the fracture toughness and fracture processes of cementitious concretes containing binders modified with a combination of two active mineral additives (i.e., FA and NS), Table 12 summarizes the most important observations from the experiments carried out. The table below includes the average values of the fracture mechanic parameters of the analyzed composites in linear and nonlinear terms. In addition, it contains characterization of the properties of the composites related to the process of formation and propagation of cracks in their structures and the moment of destruction of the tested beams subjected to mode I loading conditions.

At this point, it should also be noted that both high qualitative and quantitative convergence were observed in the obtained test results. The percentage increments of all analyzed mechanical parameters and fracture mechanic parameters oscillated at a quite similar level for all tested concretes. This is evident from their good convergence. Moreover, this was also confirmed by the low dispersion of the obtained test results and the small values for the coefficients of variation (i.e., not exceeding 10%) (Table 6, Table 7, Table 9, Table 10 and Table 11). 

## 5. Conclusions

This study analyzed the mode I fracturing of concrete composites reinforced by particles of FA in conjunction with nanoparticles of NS, which were tested after 28 days of curing. The fracture mechanic parameters of the analyzed composites in linear and nonlinear terms, as well as the fracture behavior of the concretes in question, were evaluated throughout this manuscript. The main strength parameters of the concretes in question were also evaluated.

The following conclusions were reached from the conducted research on the fracture mechanic parameters of concretes involving FA from coal combustion and synthetic NS:

(1) The inclusion of FA from coal combustion in combination with synthetic NS significantly improved the fracture mechanic parameters both in linear and non-linear terms. The values of the main strength characteristics of such concretes also clearly improved.
(2) Based on the obtained test results, it can be strongly concluded that, as in the case of the strength parameters, with regard to the fracture toughness results, the effect of the applied modification was evident. All of the three analyzed fracture mechanic parameters (i.e., ${\;K}_{Ic}^{S}$, *CTOD*_c_, and *J*_Ic_) increased significantly. In comparison with the reference concrete, they amounted to levels ranging from almost 20% for the composite of the TC-1 series (i.e., with only 5% NS), almost 30% for the concrete of the TC-2 series containing 5% NS and 25% FA, and almost 40% for the concrete with a combined addition of 5% NS and 15% FA.
(3) A rather clear qualitative convergence of the strength parameters of the composites with the fracture mechanic parameters was observed. Therefore, it can be presumed that both the strength of such materials and their fracture toughness are determined by similar processes related to modification of the structure of the cementitious matrix in ternary concretes. The improvement in the analyzed parameters resulted from the synergy of the interaction between the NS and FA. As a result of this effect, the arrangement of the concrete composite phases changed toward more homogeneous structures, as well as the activation of FA grains for the formation of additional permanent C-S-H type compounds and filling the voids in the material with products of intensified pozzolanic reactions [81]. As a consequence, this led to a more homogeneous phase system in the cementitious matrix, which resulted in its greater rigidity, higher resistance to external mechanical impacts, and lower susceptibility to the formation of intra-material defects.
(4) The substitution of the binder composition, both with the NS alone and the combination of NS and FA, influenced both the shape and appearance of the *F–CMOD* and *F–D* destruction curves, as well as the process of crack propagation during the ongoing incremental loads. The concrete based on a binder containing only NS showed signs of brittle fracturing. The addition of FA to the binder composition caused a significant change in the way the samples cracked and in the characteristics of the processes leading to the complete destruction of the beams. The concretes of the TC-2 and TC-3 series showed signs of quasi-plastic fracturing. This effect was more clearly noticeable in the material with the higher FA content.
(5) From an application point of view, the concretes containing a combination of added FA and NS, with improved fracture toughness and reduced brittleness, can be used for concrete structures in which dynamic, impact, or fatigue loads occur. These include road and bridge structures or structures in industrial construction, such as foundations for machines.

## Figures and Tables

**Table 1:** Types of nanomaterials most frequently used in concretes with FA.

Kind of Nanomaterial	Reference
nano-SiO_2_	[24]
nano-Al_2_O_3_	[25]
nano-TiO_2_	[26,27]
nano-ZnO_2_	[28]
nano-CaCO_3_	[29]
nano-Fe_2_O_3_	[26]
carbon nanotubes	[30]
C-S-H nanoseeds	[31]

**Table 2:** Arrangement of the experiments.

Factor	Levels	Responses
Quantity of OPC	100, 80, 70 (wt%)	*f*_cm_ (MPa) *f*_ctm_ (MPa) $K_{Ic}^{S}$ (MNm^−3/2^) *CTOD*_c_ (m10^−5^) *J*_Ic_ (Nm)
Quantity of FA	0, 15, 25 (wt%)	
Quantity of NS	0, 5 (wt%)	

*f*_cm_ = compressive strength, *f*_ctm_ = splitting tensile strength, $K_{Ic}^{S}$ = critical stress intensity factor, *CTOD*_c_ = critical crack tip opening displacement, and *J*_Ic_ = critical unit work of failure.

**Table 3:** Physical and chemical properties of cementitious fillers.

Description	OPC	FA	NS
Physical characteristics			
Specific gravity (g/cm^3^)	3.11	2.14	1.10
Blaine’s fineness (m^2^/g)	0.33	0.35	200
Particle diameter (μm)	40	30	0.012
Composition (%)			
Calcium oxide (CaO)	71.06	2.35	-
Silicon dioxide (SiO_2_)	15.00	55.27	>99.80
Aluminum trioxide (Al_2_O_3_)	2.78	26.72	-
Iron (III) oxide (Fe_2_O_3_)	2.72	6.66	-
Magnesium oxide (MgO)	1.38	0.81	-
Sulphur trioxide (SO_3_)	4.56	0.47	-
Potassium oxide (K_2_O)	1.21	3.01	-
Phosphorus pentoxide (P_2_O_5_)	-	1.92	-
Titanium oxide (IV) (TiO_2_)	-	1.89	-
Silver oxide (I) (Ag_2_O)	-	0.10	-
Loss on ignition	1.24	3.20	1.0

**Table 4:** The particle size distribution of the aggregates used.

Fraction (mm)	Aggregate Content Fraction (%)		
	Sand	Gravel	Mix
0–0.125	2.9	0.7	1.7
0.125–0.25	14.8	0.4	5.6
0.25–0.5	41.1	0.4	15.3
0.5–1.0	32.7	1.6	12.4
1.0–2.0	4.5	6.9	5.7
2.0–4.0	4.0	19.9	13.9
4.0–8.0	0.0	63.1	40.2
8.0–16.0	0.0	7.0	5.2
Sand point	96.0	10.0	40.7

**Table 5:** Details of mixture proportions per cubic meter.

Constituent of Concrete Mix	Mix ID			
	REF	TC-1	TC-2	TC-3
OPC (kg)	352	334.4	281.6	246
FA (kg)	0	0	52.8	88
NS (kg)	0	17.6	17.6	17.6
Water (kg)	141	141	141	141
SP (kg)	0	6	6	6
Sand (kg)	676	676	676	676
Gravel (kg)	1205	1205	1205	1205

**Table 6:** Compressive strength results.

Mix ID	$\bar{f_{cm}}$ (MPa)	Relation to REF Concrete	δ (MPa)	ν (%)	$f_{c,max.}$ (MPa)	$f_{c,min.}$ (MPa)
REF	38.32	100%	2.87	5.8	41.07	35.44
TC-1	45.30	118.22	3.08	6.4	48,32	42.25
TC-2	52.76	137.68	3.54	7.3	56.29	49.23
TC-3	49.45	129.04	3.82	7.7	53.26	45.62

**Table 7:** Splitting tensile strength results.

Mix ID	$\bar{f_{ctm}}$ (MPa)	Relation to REF Concrete	δ (MPa)	ν (%)	$f_{ct,max.}$ (MPa)	$f_{ct,min.}$ (MPa)
REF	2.90	100%	0.18	6.7	3.07	2.69
TC-1	3.38	116.55	0.21	6.9	3,60	3.16
TC-2	3.95	136.21	0.23	7.4	4.19	3.70
TC-3	3.72	128.28	0.27	7.9	4.02	3.44

**Table 8:** Significant features of *F*–*CMOD* and *F–D* curves observed during the conducted experiments for all types of concrete.

Mix ID	The Analyzed Feature of the *F*–*CMOD* and *F*–*D* Curves				
	Force *F*_max_ (kN)	*CMOD* at Beam Failure (mm)	Behavior in Fracture Process	Number of Load Cycles (pcs)	The Relative Length of the Fracture Process
REF	Approximately 3.0	1.0	Slightly quasi-plastic	13–15	Short
TC-1	Approximately 3.5	0.6	Clearly brittle	12–14	Extremely short
TC-2	Approximately 4.2	1.1	Slightly quasi-plastic	14–16	Slightly elongated
TC-3	Approximately 3.8	1.3	Clearly quasi-plastic	15–18	Clearly elongated

**Table 9:** Critical stress intensity factor results.

Mix ID	$\bar{K_{Ic}^{S}}$ (MNm^−3/2^)	Relation to REF Concrete	δ (MNm^−3/2^)	ν (%)	$K_{Ic}^{S},max.$(MNm^−3/2^)	$K_{Ic}^{S},min.$(MNm^−3/2^)
REF	1.06	100%	0.06	7.3	1.13	0.99
TC-1	1.26	118.87	0.08	7.8	1.34	1.17
TC-2	1.46	137.74	0.10	8.6	1.57	1.35
TC-3	1.37	129.25	0.11	9.1	1.48	1.25

**Table 10:** Critical crack tip opening displacement results.

Mix ID	$\bar{{CTOD}_{c}}$ (m10^−5^)	Relation to REF Concrete	δ (MNm^−3/2^)	ν (%)	${CTOD}_{c},max.$(m10^−5^)	${CTOD}_{c},min.$(m10^−5^)
REF	1.041	100%	0.08	7.6	1.122	0.962
TC-1	1.243	119.40	0.10	8.3	1.239	1.144
TC-2	1.436	137.94	0.12	9.2	1.557	1.315
TC-3	1.357	130.36	0.13	9.6	1.486	1.225

**Table 11:** Critical unit work of failure results.

Mix ID	$\bar{J_{Ic}}$ (Nm)	Relation to REF Concrete	δ (Nm)	ν (%)	$J_{Ic},max.$(Nm)	$J_{Ic},min.$(Nm)
REF	48.51	100%	3.64	7.5	52.16	44.86
TC-1	57.43	118.39	4.82	8.4	62.24	52.61
TC-2	66.12	136.30	6.15	9.3	72.27	59.98
TC-3	62.30	128.42	5.92	9.5	68.25	56.44

**Table 12:** Characterization of composites incorporating FA and NS with respect to the study of their fracture toughness and fracture processes.

Analyzed Parameter	Mix ID			
	REF	TC-1	TC-2	TC-3
$\bar{K_{Ic}^{S}}$ (MNm^−3/2^)	1.06	1.26	1.46	1.37
$\bar{{CTOD}_{c}}$ (m10^−5^)	1.041	1.243	1.436	1.357
$\bar{J_{Ic}}$ (Nm)	48.51	57.43	66.12	62.30
Fracture process type	Slightly quasi-plastic	Clearly brittle	Slightly quasi-plastic	Clearly quasi-plastic
Fracture process length	Short and stable	Short and sudden	Elongated and slow	Strongly elongated and extremely slow
Shape of *F*–*CMOD* and *F*–*D* curves	Slightly inclined	Slender	Slightly inclined with few disturbances	Strongly sloped with numerous disturbances

## Data Availability

Data are contained within the article.
